# Fibrin Fiber Stiffness Is Strongly Affected by Fiber Diameter, but Not by Fibrinogen Glycation

**DOI:** 10.1016/j.bpj.2016.02.021

**Published:** 2016-03-29

**Authors:** Wei Li, Justin Sigley, Marlien Pieters, Christine Carlisle Helms, Chandrasekaran Nagaswami, John W. Weisel, Martin Guthold

**Affiliations:** 1 Department of Physics, Wake Forest University, Winston-Salem, North Carolina; 2 Centre of Excellence for Nutrition, North-West University, Potchefstroom, South Africa; 3 Department of Physics, University of Richmond, Richmond, Virginia; 4 Department of Cell and Developmental Biology, University of Pennsylvania School of Medicine, Philadelphia, Pennsylvania

## Abstract

The major structural component of a blood clot is a mesh of fibrin fibers. Our goal was to determine whether fibrinogen glycation and fibrin fiber diameter have an effect on the mechanical properties of single fibrin fibers. We used a combined atomic force microscopy/fluorescence microscopy technique to determine the mechanical properties of individual fibrin fibers formed from blood plasma. Blood samples were taken from uncontrolled diabetic patients as well as age-, gender-, and body-mass-index-matched healthy individuals. The patients then underwent treatment to control blood glucose levels before end blood samples were taken. The fibrinogen glycation of the diabetic patients was reduced from 8.8 to 5.0 mol glucose/mol fibrinogen, and the healthy individuals had a mean fibrinogen glycation of 4.0 mol glucose/mol fibrinogen. We found that fibrinogen glycation had no significant systematic effect on single-fiber modulus, extensibility, or stress relaxation times. However, we did find that the fiber modulus, *Y*, strongly decreases with increasing fiber diameter, *D*, as $Y\propto D^{-1.6}$. Thin fibers can be 100 times stiffer than thick fibers. This is unusual because the modulus is a material constant and should not depend on the sample dimensions (diameter) for homogeneous materials. Our finding, therefore, implies that fibrin fibers do not have a homogeneous cross section of uniformly connected protofibrils, as is commonly thought. Instead, the density of protofibril connections, *ρ*_Pb_, strongly decreases with increasing diameter, as $\rho_{\text{Pb}}\propto D^{-1.6}$. Thin fibers are denser and/or have more strongly connected protofibrils than thick fibers. This implies that it is easier to dissolve clots that consist of fewer thick fibers than those that consist of many thin fibers, which is consistent with experimental and clinical observations.

## Introduction

**Figure 1:**
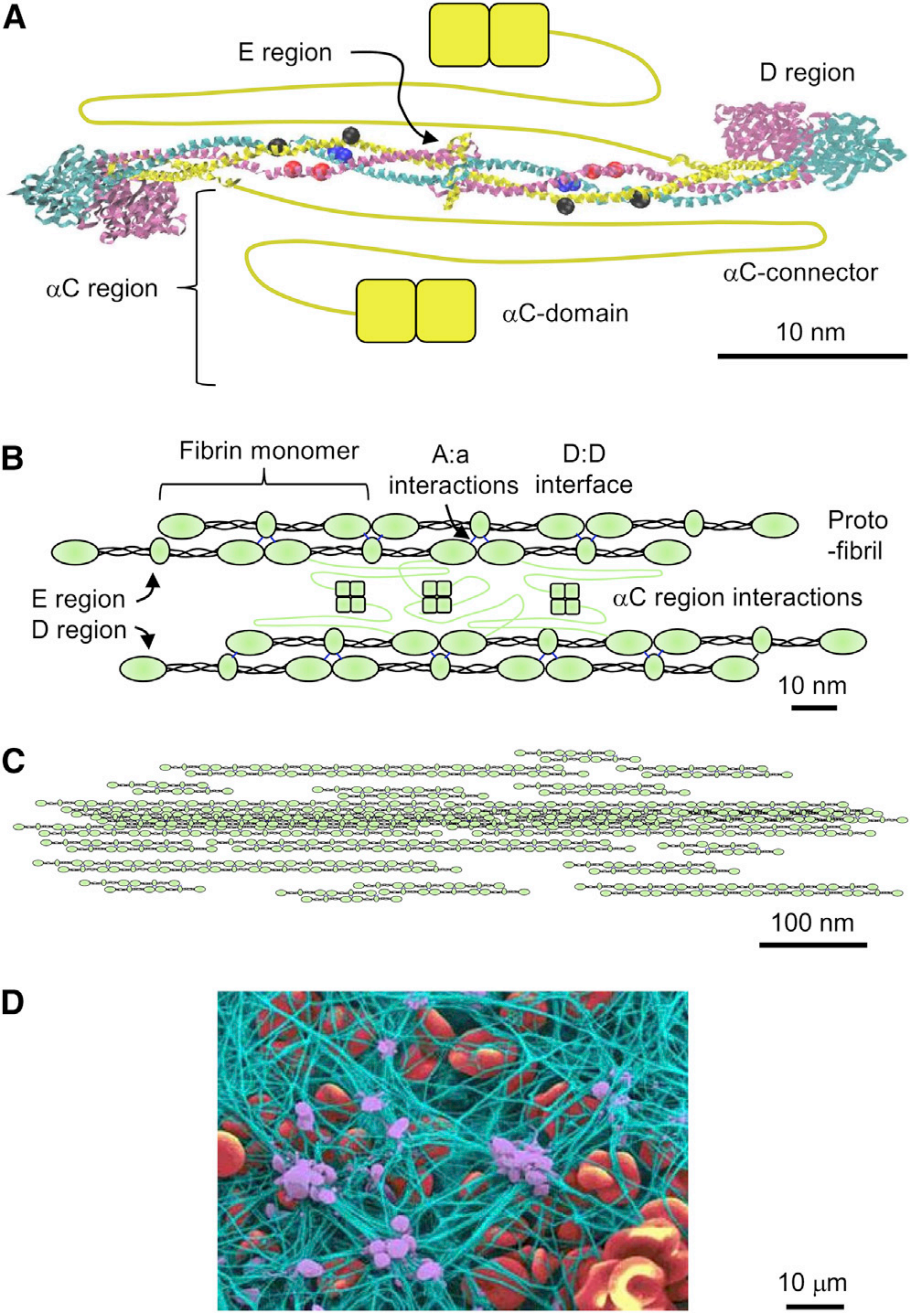
Fibrin fiber assembly. (*A*) Crystal structure of human fibrinogen (42). Fibrinogen has a nearly centrosymmetric structure consisting of two A*α*-chains (*yellow*, 610 amino acids), two B*β*-chains (*magenta*, 461 amino acids), and two *γ*-chains (*cyan*, 411 amino acids). Not resolved in the crystal structure are residues *α*1–26 and *α*201–610, *β*1–57 and *β*459–461, and *γ*1–13 and *γ*395–411. The central E region, which contains the N-termini of all chains, including fibrinopeptides A (*α*1–16) and B (*β*1–14), is connected by two triple-helical coiled coils to the two distal D regions. The *α*-C regions (*α*221–610), which are drawn in by hand, consist of the unstructured, 61-nm-long *α*C connector (*α*221–391; drawn as a *yellow line*) and the folded *α*C domain (*α*392–610; drawn as two *yellow squares*). The small blue, red, and black spheres indicate plasmin lysis sites in the coiled coils; numerous additional lysis sites (not shown) are in the *α*-C region. (*B*) Fibrin interactions. The half-staggered assembly of fibrin monomers into double-stranded protofibrils is mainly mediated by A:a knob-hole interactions and D:D interface interactions, and to a lesser extent by B:b knob-hole interactions (not shown). Lateral (radial) assembly of protofibrils into mature fibers is thought to be mostly mediated by interactions of the *α*-C regions, resulting in a dense, complex network of *α*-C regions between protofibrils. For clarity, not all *α*-C regions are shown in the space between protofibrils. (*C*) Schematic, longitudinal cross section of a mature, ∼130-nm-wide fibrin fiber, showing a dense fiber core and a less dense fiber periphery. This decrease in fiber density (or more specifically, bond density) with increasing fiber diameter is one of the key findings of this study. The lateral structure is mostly held together by the network of connected *α*-C regions (partially shown in *B*, but for clarity not shown in *C*). (*D*) A false-color scanning electron micrograph of a blood clot conveys the central role of fibrin fibers in providing mechanical and structural support to a blood clot. Green, fibrin fibers; purple, platelets; red, red blood cell (image courtesy of Y. Veklich and J. W. Weisel, University of Pennsylvania School of Medicine). To see this figure in color, go online.

In the event of injury to a blood vessel, platelets aggregate at the injury site and the clotting cascade is initiated to form a blood clot that will stop blood flow through the injured blood vessel. The clotting cascade is a complex series of protein activations culminating in the activation of fibrinogen by activated thrombin. Fibrinogen is an abundant blood protein that consists of a central, globular E region and two distal D regions that are connected by two triple *α*-helical coiled coils (Fig. 1). Thrombin proteolytically removes two fibrinopeptides A and two fibrinopeptides B from the N-termini of the *α*- and *β*-chains in the central E region, thereby exposing two knobs “A” (Gly-Pro-Arg) on the *α*-chain and then two knobs “B” (Gly-His-Arg) on the *β*-chain, and thus converting fibrinogen to fibrin. Fibrin monomers (45 nm long and 4.5 nm thick) bind to each other in a half-staggered fashion to form double-stranded protofibrils. The key interactions for protofibril formation are the A:a and B:b knob-hole interactions, in which the charged knobs “A” and knobs “B” bind to holes “a” and holes “b” in the distal D region, and the D:D interactions of abutting D regions (Fig. 1). Protofibrils aggregate laterally (radially) to form the mature, ∼130-nm-thick fibrin fibers of a blood clot. Lateral aggregation of protofibrils is poorly understood, but there is evidence that interactions between the long, largely unstructured *α*-C regions play a key role (1, 2) in lateral aggregation.

The main physiological function of blood clots, which mainly consist of platelets and a meshwork of microscopic fibrin fibers, is to stem the flow of blood. Since this is a mechanical task, studies have been conducted over the past 60 years to elucidate the mechanical properties of clots. The vast majority of these studies were done on whole clots. For example, rheometry techniques were used to determine the loss modulus and storage modulus and other mechanical properties of clots (3, 4), and it was found that these properties can be associated with thrombotic disease (5, 6, 7, 8). In a clinical setting, thromboelastography is used by clinicians to uncover clotting abnormalities and to reduce risk in surgeries (9, 10). In an effort to deepen our understanding of clot behavior, new techniques, such as atomic force microscopy (AFM) and optical traps, have been developed in recent years to determine the mechanical properties of single fibrin fibers within a fibrin clot (11, 12, 13). Single-fiber experiments bridge the gap in scale between whole-clot experiments and molecular experiments, and the data from single-fiber experiments are a key building block in constructing realistic models of fibrin clots (14). Single-fiber data are also used to test the predictions of molecular-dynamics simulations at the molecular level (15, 16, 17).

Most single-fiber investigations have been done on clots formed from purified fibrinogen, and few data are available regarding the mechanical properties of individual fibrin fibers in plasma clots, which are more physiological and quite different in structure and properties (13). The use of plasma clots would make it possible to directly measure the properties of pathophysiological clots from patients who suffer from specific clotting disorders and diseases. To our knowledge, the work presented here is the first single-fiber study to use plasma clots from patients.

Diabetes is a risk factor for cardiovascular disease (CVD), increasing CVD risk by 2–4 times (18), and 68% of morbidity in diabetic patients is due to CVD (19). According to the Centers for Disease Control and Prevention, diabetes affects 25.8 million people, or 8.3% of the population, in the United States (19). The relationship between CVD and diabetes is not well understood, although alterations to the properties of fibrin clots in diabetic patients have been reported. Several studies have examined clots formed from plasma or fibrinogen isolated from diabetic patients, as well as fibrinogen glycated in the lab, in an attempt to understand the connections between CVD and diabetes. However, many of these studies reported conflicting findings, likely as a result of differences in the study design (e.g., plasma versus purified fibrinogen, and use of diabetic versus normal plasma with added glucose), study population, and analytical techniques and methods used. An important plausible mechanism for altered fibrin networks in diabetic patients is nonenzymatic glycation of fibrinogen in the presence of uncontrolled blood glucose levels. A few studies reported increased resistance to fibrinolysis in samples with increased glycation (18, 20). Some studies showed a shorter lag phase in polymerization and decreased permeability in diabetic clots (20, 21), whereas a few other studies showed no difference in polymerization, kinetics, clot porosity, and clot density in diabetic samples compared with control samples (22, 23). No studies investigating single-fiber properties in clots formed from the plasma of diabetic patients have been reported to date.

In this investigation, we determined the mechanical properties of individual fibrin fibers formed from the plasma of diabetic patients and healthy controls. Our goal was to investigate whether altered fibrin clot properties in diabetic patients are related to effects of protein glycation on single-fiber properties. Our results show that increased glycation does not alter the modulus or extensibility of single fibrin fibers in a predictable way. Thus, we conclude that glycation does not have a direct effect on single-fiber mechanical properties, and the negative effects of diabetes on cardiovascular health likely have a different origin than altered single-fiber mechanical properties.

Because of the large variation in fiber size in our experiments, we also investigated the effect of fiber diameter. We found that the stiffness (modulus) of fibrin fibers, *Y*, strongly decreased with increasing fiber diameter, *D*, in all samples. *Y* scaled as *D*^−1.6^ over a tested diameter range of ∼20–400 nm, and the thinnest fibers were >100 times stiffer than the thickest fibers. This is very unusual behavior since the modulus is a material constant and does not depend on the dimensions (diameter) of a material, assuming the material has a regular, homogeneous cross section. Our finding, therefore, implies that fibrin fibers do not have a homogeneous cross section of uniformly connected protofibrils, as is commonly thought. Instead, the density of protofibril connections, *ρ*_Pb_, strongly decreases with increasing diameter, as $\rho_{\text{Pb}}\propto D^{-1.6}$. Thin fibers are much denser and/or have more densely or strongly connected protofibrils than thick fibers. We propose, to our knowledge, a new fibrin fiber model in which fibers are less densely connected at increasing diameters, resulting in a lower modulus. We corroborate this model with measurements obtained from individual fibers in plasma fibrin clots as well as purified fibrin clots.

This finding has several important implications. Although the assembly of fibrin monomers into protofibrils via A:a and B:b knob-hole interactions and D:D interface interactions is relatively well understood, the lateral assembly of protofibrils into mature fibers is poorly understood, and models for the lateral cross section of a fiber are only speculative. This lateral assembly is one of the critical pieces that are still missing from a full understanding of clot formation. Our data provide information about the lateral cross-sectional organization of fibrin fibers, and therefore contribute to our understanding of fibrin fiber formation. Models for fibrin fiber lateral assembly need to be modified to account for this decreasing lateral density. It is also important to note that any parameter that affects the radius of a fiber, such as the fibrinogen or thrombin concentration, will strongly affect the modulus of the fiber and thus the entire clot. Thin fibers are denser than thick fibers, and thick fibers likely have a denser core and a less dense periphery. Thus, thin fibers and the core of thick fibers are likely harder to dissolve. This key finding may explain the clinically found relationship between thin fibers and increased risk of thrombotic diseases, in particular, myocardial infarction, ischemic stroke, and venous thromboembolism (7).

## Materials and Methods

### Plasma collection and determination of fibrinogen concentration and glycation

We investigated the fibrin fibers of plasma clots formed from 14 different plasma samples and obtained ∼15 measurements per sample for all experiments. The samples included four control (nondiabetic) individuals, five controlled diabetic patients, and five noncontrolled diabetic patients. Blood was collected from the diabetic patients both before intervention (uncontrolled diabetics) and after intervention (controlled diabetics). Several clots were formed from the same plasma samples over the duration of the study. We did not observe any significant difference in the viscoelastic properties of fibers from the same plasma sample over the course of the investigation. All of the plasma samples were collected from black females between the ages of 44 and 65, with a median age of 58. To limit the number of variables, we did not include males.

To determine whether glycation had an effect on the mechanical properties of single fibrin fibers, we recruited 20 type 2 diabetic and 18 nondiabetic individuals (control) (20, 23). From this group, we used the subset of 14 samples mentioned above for single-fiber studies. Patients had to be uncontrolled (HbA1C > 9%) on maximum-dose combination oral hypoglycemic medication, have a body mass index (BMI) of >25 kg/m^2^, be 40–65 years of age, and have sufficiently controlled blood pressure (<140/90 mm Hg) to be included in the study. After baseline blood samples were collected, the patients underwent a three-step intervention program. First, the patients were taught how to monitor glucose levels, coordinate insulin use with meals, and manage hypoglycemic events with glucagon. Second, the patients received 10 IU (equivalent to 0.347 mg) of insulin daily in addition to the current treatment of maximum-dose oral hypoglycemic treatment. Metformin use was unchanged, sulphonylureas were stopped, and insulin use was adjusted individually until four out of five subsequent fasting blood glucose values were <7.2 mM. Lastly, short-acting insulin was used to control postprandial glucose levels (<10 mM). Once both fasting and postprandial glycemic control were achieved, the subjects remained on treatment for 8 days before end blood samples were collected. Nondiabetic control subjects with matching age, gender, and BMI were included. Baseline oral glucose tolerance tests were done to rule out diabetes in the controls. Citrated blood was collected from patients. Within 30 min of collection, the blood was centrifuged for 15 min at 2000 *g* at 4°C. The plasma was extracted, snap-frozen, and stored at −80°C until fiber samples were prepared.

### Fibrinogen concentration

Fibrinogen concentration was measured as previously described in (23) using a modified Clauss method on an Automated Coagulation Laboratory 200 (Instrumentation Laboratories, Milan, Italy; between-run coefficient of variation (CV) = 3%). Fibrinogen was purified from the plasma of each subject by IF-1 affinity chromatography as described in (24). Purified fibrinogen was run on 10% SDS-PAGE gels to confirm purity and the absence of degradation of the fibrinogen preparations (see Fig. 1 in (23)).

### Fibrinogen glycation

Fibrinogen glycation was measured as previously described (23) using a two-reagent enzymatic assay (GlyPro assay, Genzyme Diagnostics, Cambridge, MA; between-run CV = 5%). This is a specific enzymatic method for the direct measurement of glycated proteins in serum or plasma. The first reagent digests the proteins and subsequently releases glycated protein fragments. Ketoamine oxidase in the second reagent facilitates the specific oxidation of the ketoamine bond of the glycated protein fragment substrate. Liberation of hydrogen peroxide allows a colorimetric determination of the amount of glycated protein in an end-point reaction. Absorbance at 550 nm was measured after the addition of reagent 1 and again after reagent 2. Results were calculated as follows: glycated protein (*μ*M) = ((Δ*A*_sample_)/(Δ*A*_calibrator_)) × calibrator value (*μ*M).

### Substrate preparation

Striated cover slides were prepared as previously described (11, 12). Briefly, optical adhesive (NOA-81, Norland Products, Cranbury, NJ) was placed on a cover slide. A rectangular polydimethylsiloxane stamp was pressed into the adhesive to create a 1.5 cm × 1.5 cm wide and 0.5 cm deep well to hold the buffer. In the center of the well, a drop of optical glue was placed and a second polydimethylsiloxane stamp was used to create a striated surface with 6.5 *μ*m wide ridges, and 13.5 *μ*m wide and 6.0 *μ*m deep grooves. The optical glue was then cured under 365 nm UV light (3UV transilluminator, UVP, Upland, CA) for 1.5 min.

### Fibrin sample preparation

All chemicals were obtained from Sigma-Aldrich unless otherwise noted. To form fibrin fibers, an 18 *μ*L aliquot of plasma solution (14 *μ*L of citrated plasma and 4 *μ*L of 0.1 M CaCl_2_) was combined with 2 *μ*L of thrombin (Enzyme Research Laboratories, South Bend, IN; final concentration 0.1 NIH units/mL) and pipetted onto the striated cover slide. The clotting reaction ran for 1 h in a moist atmosphere at room temperature. This time period was chosen to allow completion of fiber formation, including stabilization of fibrin by factor XIIIa. After an hour, the slide was rinsed with calcium-free buffer (140 mM NaCl, 10 mM Hepes, pH 7.4). A pipette tip was used to manually remove excess fibers from the top of the sample. The sample was then rinsed with fibrin buffer (140 mM NaCl, 10 mM Hepes, 5 mM CaCl_2_, pH 7.4). The fibers were labeled with 20 nm carboxyl-coated fluorospheres (Invitrogen, Carlsbad, CA) and rinsed with fibrin buffer once again.

Purified fibrin samples were formed in a manner similar to that used for the plasma fibrin samples. In this case, a 2 *μ*L mixture of thrombin (final concentration 0.3 NIH units/mL) and FXIII (Enzyme Research Laboratories, South Bend, IN; final concentration 9 Loewy units/mL) was added to 18 *μ*L of purified fibrinogen in concentrations varying from 0.8 mg/mL to 6 mg/mL. After clotting, the mixtures were rinsed with calcium-free buffer and labeled with 20 nm fluorospheres.

### Manipulations

**Figure 2:**
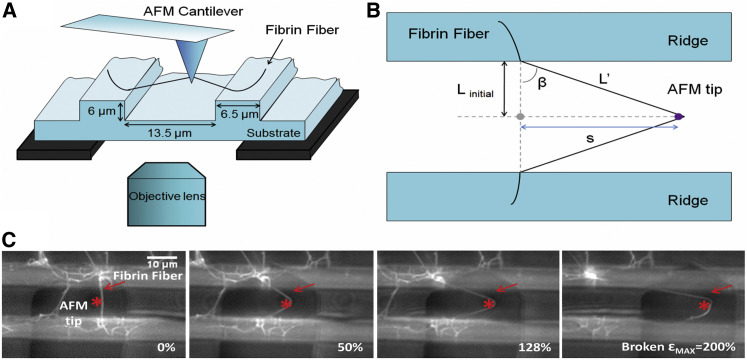
Fibrin fiber manipulation. (*A*) Schematic of fibrin fiber manipulation. The fiber is suspended over the grooves in a striated substrate. The AFM tip, located above the sample, pulls on the fiber while the optical microscope, located below the sample, acquires images and movies of the manipulation. (*B*) Top view schematic of fiber manipulation. *L*_initial_ is half the initial length of the fiber, *L*′ is half the length of the stretched fiber, and *s* is the distance the tip has traveled. *L*′ can be found trigonometrically from *L*_initial_ and *s*, and the strain can be calculated from these quantities (see text). Schematics (*A*) and (*B*) were adapted from (11). (*C*) Optical microscopy movie frames of a fiber being stretched and broken. The movie was recorded from underneath the sample. The large dark object (*rectangle plus triangle shape*) is the AFM cantilever and the AFM tip is marked by an *asterisk*. The fiber broke at a strain of 200%. Scale bar, 10 *μ*m. To see this figure in color, go online.

Fibrin clots were visualized with an inverted fluorescence microscope (Fig. 2). Straight fibers that spanned a groove and had no branch points within that span were chosen for manipulation. The groove width corresponds approximately to the length between branch points in a plasma fibrin clot (5, 13). Fiber modulus, extensibility, and stress relaxation behavior were determined as previously described (11); for details, see Supporting Materials and Methods in the Supporting Material. Manipulations were performed using a combined AFM (Topometrix Explorer, Veeco Instruments, Woodbury, NY) and inverted fluorescence microscope (Axiovert 200 or Observer D, Zeiss, Göttingen, Germany). The fiber sample was placed between the AFM and optical microscope using a customized stage that allowed the sample to be moved independently of either microscope. Illumination of the sample was provided by a camera light in the AFM. Fibers were stretched with the AFM probe (CSC-38, MikroMash, Willsonville, OR) at a rate of ∼320 nm/s. Cantilever deflection, distance, and time data were collected with the use of NanoManipulator software (3rd Tech, Chapel Hill, NC). Images were collected during manipulations using a Zeiss AxioCam and Zeiss Axiovert software, or a Hamamatsu EM-CCD C9100 camera (Hamamatsu Photonics, KK, Japan) with IPLab software (Scanalytics, Fairfax, VA). Fiber diameter, *D*, was determined by using the AFM to image the fiber on top of the ridge, adjacent to where the fiber was manipulated. The fiber cross section was calculated assuming a circular cross section, $A=\pi {\left(D/2\right)}^{2}$.

#### Extensibility

In an extensibility measurement, a fiber is simply stretched until it breaks, and the strain at the breaking point, *ε*_max_ = Δ*L*_max_/*L*_inital_, is termed the extensibility. Strain is defined as *ε* = Δ*L*/*L*_inital_, with Δ*L* = *L'* − *L*_initial_, where *L*_initial_ is the initial length of the fiber and *L'* is the extended length of the fiber.

#### Stiffness (stretch modulus) and relaxation times

We used incremental stress-strain (force-extension) curves to determine the longitudinal stiffness (modulus) and stress relaxation times of fibrin fibers.

In a simple stress-strain curve, a force, *F*, is applied longitudinally to an elastic fiber with cross-sectional area, *A*, causing a strain, *ε*. The stretch modulus, *Y*, is the proportionality constant between the applied stress, *σ* = *F*/*A*, and the resulting strain, *ε*: *σ* = *Y* × *ε*. An example of an incremental stress-strain curve is shown in Fig. S1. In these measurements, the fiber is stretched and then held at a constant strain for a period of time before being stretched again. The process is repeated at higher and higher strains. The slope of the unrelaxed stress-strain curve corresponds to the total stretch modulus (total stiffness), and the slope of the relaxed stress-strain curve corresponds to the elastic component of the total modulus. We found that the elastic modulus is typically a factor of ∼0.6 lower than the total modulus, and for simplicity, we will only report the total modulus here (for the elastic modulus, see Supporting Materials and Methods). When the fiber is held at constant strain, the stress decays (see Supporting Materials and Methods). This is indicative of viscoelastic behavior. The simplest mechanical model that can account for these observations (the two relaxation rates; stress does not decay to zero) is a generalized Kelvin model consisting of an elastic spring with modulus *Y*_∞_, in parallel with two Maxwell elements consisting of a dashpot and a spring in series (11). For this model, the equation for stress relaxation becomes

(1) $$\sigma \left(t\right)=\varepsilon_{0}\left[Y_{\infty }+Y_{1}\times e^{-t/\tau_{1}}+Y_{2}\times e^{-t/\tau_{2}}\right],$$

where *Y*_∞_ is the relaxed elastic modulus and *Y* is the total elastic modulus, *Y* = *Y*_∞_ +*Y*_1_ + *Y*_2_. By fitting an exponential curve to the stress decay, we can determine key mechanical properties (i.e., the total modulus, elastic modulus, and relaxation times) of the fibers (11). A double exponential (two relaxation times) is required to obtain good fits (see Supporting Materials and Methods for a comparison between a single exponential and a double exponential). Thus, applying this Kelvin model to fibrin fibers yields several parameters. *Y*_1_ and *Y*_2_, which are the spring constants of spring 1 and spring 2, can be obtained from the fits. Additionally, the relaxation times *τ*_1_ and *τ*_2_ can also be obtained from the fits. Moreover, the relaxation times, the elastic modulus of the springs, and the viscosity of the dashpot elements are related via *τ*_1_ = *μ*_1_/*Y*_1_ and *τ*_2_ = *μ*_2_/*Y*_2_, and therefore the viscosities *μ*_1_ and *μ*_2_ of the dashpots can also be extracted from our data. The total modulus, *Y*, and the extensibility *ε*_max_ are reported in the main text since they are model independent, and *Y*_∞_ (also model independent) and *Y*_1_, *Y*_2_, *τ*_1_, *τ*_2_, *μ*_1_, and *μ*_2_ (all model dependent) are reported in Supporting Materials and Methods. We refrained from assigning any of the elements in the Kelvin model to actual structural elements in single fibers because that would be too speculative. Such assignments would require modifying some structural elements in the actual fibrin fiber (e.g., removing the *α*-C region or other bonds) and then testing the effect on the fibrin fiber mechanical properties.

### Statistical analysis

Means and standard deviations were calculated using standard equations. To determine statistical significance between samples, a two-tailed *t*-test was used with an *α*-level set at 0.05. Linear and monotonic relationships between two variables were tested using Pearson’s correlation and Spearman’s correlation. Detailed results of the statistical analysis are reported in Supporting Materials and Methods.

## Results

### Fiber viscoelastic properties

Fibrin fibers of plasma clots from 14 different plasma samples, as detailed in the Materials and Methods section, were investigated. The blood fibrinogen concentration in the four control and five diabetic patients ranged from 3.5 to 5.6 mg/mL. Fibrinogen glycation ranged from 3.0 to 11.8 mol glucose/mol fibrinogen. Before intervention, the uncontrolled diabetic patients had an average fibrinogen glycation of 8.8 ± 3.4 mol glucose/mol fibrinogen, which decreased to an average of 5.0 ± 2.4 after intervention. The glycation of all diabetic patients decreased after intervention. The nondiabetic control group had an average fibrinogen glycation of 4.0 ± 1.0.

We determined several key mechanical properties of plasma fibrin fibers (extensibility, total modulus, and stress relaxation times (main text), and elastic modulus and viscosities (Supporting Materials and Methods)) as a function of glycation, fibrinogen concentration, and fiber diameter.

#### Glycation

**Figure 3:**
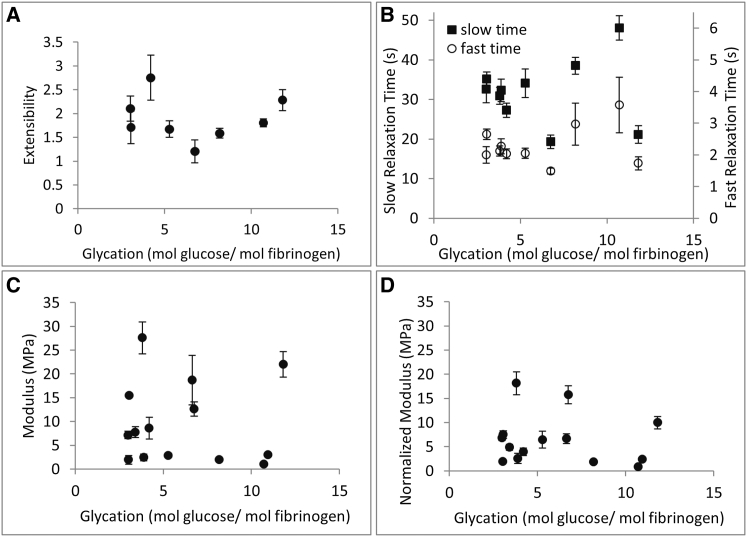
Fibrin fiber mechanical properties as a function of glycation. (*A*) Extensibility. (*B*) Fast and slow stress relaxation times. (*C*) Total modulus. (*D*) Diameter-normalized total modulus for an average 130 nm fibrin fiber. No significant, systematic trend is observed between these mechanical properties and glycation. Error bars are standard error of the mean.

Fiber extensibility for the various plasma samples from diabetic and control subjects varied from 1.2 to 2.7, largely in agreement with previously reported values for purified fibrinogen samples (11, 12). There was no clear trend in a plot of extensibility versus glycation (Fig. 3 *A*). The total modulus of the fibrin fibers varied from 1.0 MPa to 28 MPa (Fig. 3 *C*). Since the modulus of fibrin fibers varies strongly with fiber diameter as *Y*(*D*) = *Y*_0_ × *D*^−1.6^ (see below, Fig. 5 *A*), to properly compare the moduli of different fiber samples with each other, we had to adjust the modulus for this diameter dependence. Therefore, we calculated a diameter-normalized modulus, $Y_{130}^{n}$, for each fiber sample. We first multiplied each individual fiber data point by *D*^+1.6^ to determine *Y*_0_, and then we multiplied this value by 130^−1.6^ because the average fiber in each group was 130 nm (Fig. S4). We averaged these values to obtain the diameter-normalized, average modulus for a standard 130 nm fiber, $Y_{130}^{n}$, for each sample. When we plotted the normalized modulus, $Y_{130}^{n}$, versus glycation, we again observed no apparent trend (Fig. 3 *D*). When we measured stress decay, we found that the fibers had an average fast relaxation time of *τ*_1_ = 2.3 s and an average slow relaxation time of *τ*_2_ = 34 s (Fig. 3 *B*). *τ*_1_ and *τ*_2_ also did not show a dependence on glycation. In a related published work (20), it was shown that glycation did not seem to affect the diameter distribution of fibrin fibers, other than a slightly higher fraction of larger fibers in the end-point diabetic sample (after glycemic control). Therefore, it is also unlikely that glycation would indirectly affect single fibrin fiber mechanical properties through a change in diameter. In summary, glycation does not seem to have an effect on the mechanical properties of single fibrin fibers (Fig. 3).

#### Fibrinogen concentration

**Figure 4:**
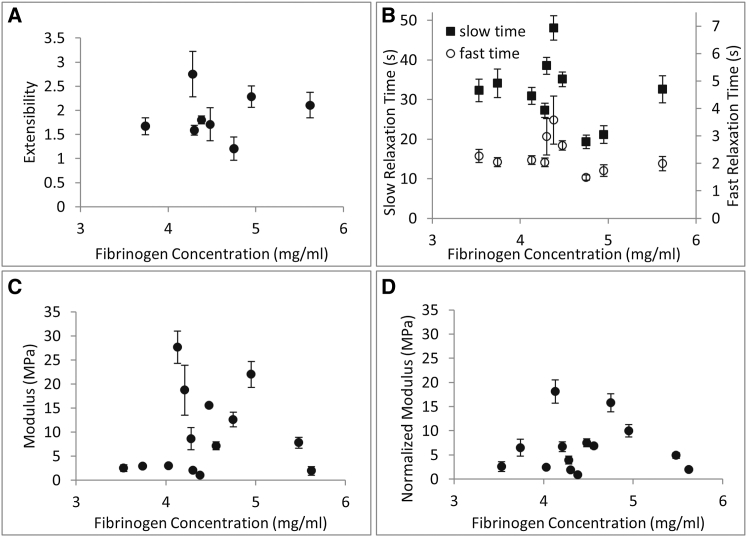
Fibrin fiber properties as a function of fibrinogen concentration. (*A*) Extensibility. (*B*) Fast and slow stress relaxation time. (*C*) Total modulus. (*D*) Diameter-normalized total modulus for an average 130 nm fibrin fiber. No significant, systematic trend is observed between these mechanical properties and fibrinogen concentration. Error bars are standard error of the mean.

Numerous clinical and epidemiological studies have indicated that an elevated fibrinogen concentration may be a risk factor for CVD (25). Using our plasma samples, we also tested whether fibrinogen concentration (in our available range from 3.5 mg/mL to 5.6 mg/mL) had an effect on single fibrin fiber properties. All of our samples had relatively high fibrinogen concentrations, consistent with studies that reported higher fibrinogen levels in black African and African-American individuals as compared with the range of 1.5–3 mg/mL typically found in healthy white individuals (26, 27). Fibrinogen concentration did not have a consistent, systematic effect on fibrin fiber extensibility, relaxation time, modulus, or diameter-normalized modulus (Fig. 4, *A–D*) at the concentrations we tested.

#### Fiber diameter

**Figure 5:**
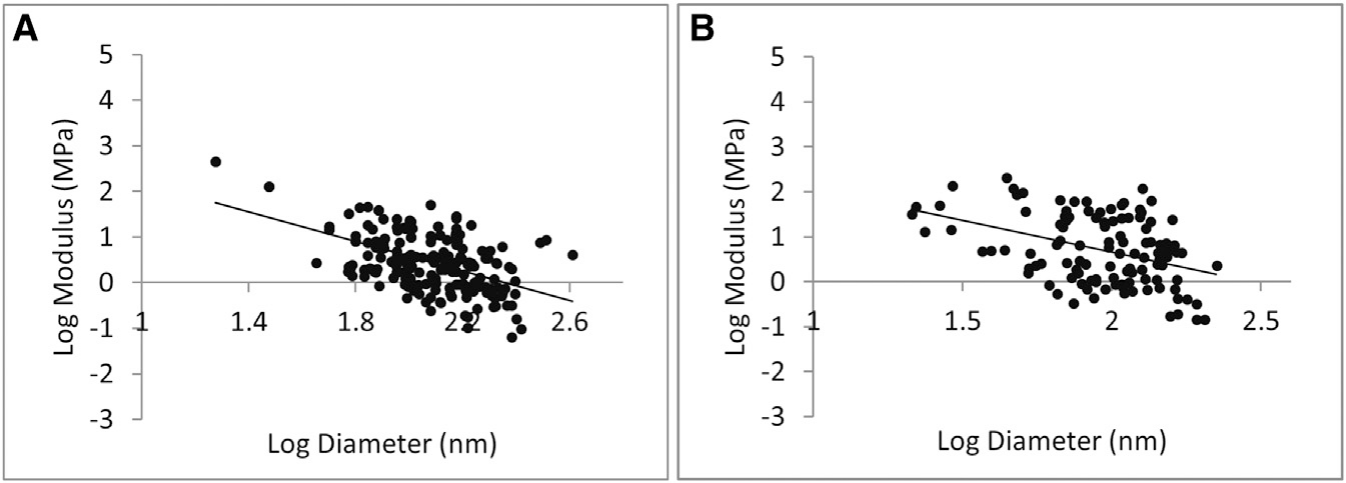
Fibrin fiber modulus as a function of fiber diameter. (*A* and *B*) Log-log plot of the total stretch modulus as a function of diameter for plasma samples (*A*) and purified fibrinogen samples (*B*). The modulus strongly depends on the fiber diameter, as $Y\propto D^{-1.6}$ (plasma) and $Y\propto D^{-1.4}$ (purified fibrinogen).

Experimental and clinical evidence indicates that fibrin clots composed of highly branched networks with thin fibers are associated with thrombotic disease (5, 6, 7, 8). However, the underlying reason for this association is not known. Therefore, we decided to also investigate the direct effect of fiber diameter on the fiber modulus. Fig. 5 shows the results of this analysis: the fiber modulus strongly decreases with increasing radius for all plasma samples and for samples formed from purified fibrinogen. There is a significant (*p* < 0.01) negative power law relationship between the fiber modulus and fiber diameter (notice the log scaling). The exponent for plasma fibrinogen fibers is −1.6 (*N* = 213, R^2^ = 0.26), whereas the exponent for purified fibrin fibers is −1.4 (*N* = 116, R^2^ = 0.17); that is, the modulus, *Y*, depends on the diameter, *D*, as $Y\propto D^{-1.6}$ (plasma) and as $Y\propto D^{-1.4}$ (purified fibrinogen). Thus, two plasma fibers with different diameters, *D*_1_ and *D*_2_, would have a different modulus by a factor of (*D*_1_/*D*_2_)^−1.6^. This is very unusual since the modulus is a material property and should not depend on fiber dimensions. As explained in more detail in the Discussion, our data imply that fibrin fibers do not have a homogeneous cross section, since for a homogeneous cross section the modulus would be independent of the diameter.

## Discussion

We set out to measure the mechanical properties of single fibrin fibers in clots formed from the plasma of diabetic patients to determine whether fibrinogen glycation has an effect on fiber mechanical properties. Our work resulted in three major overall findings. 1) Our combined AFM/inverted optical microscopy technique is suitable for testing complex plasma samples, in addition to the samples formed from purified fibrinogen that have been tested in the past. This opens up the possibility of investigating the properties of single fibrin fibers from many different patient samples. 2) We found that there was no significant direct correlation between fibrinogen glycation and fibrin fiber extensibility, modulus, and stress relaxation, as tested using Pearson’s correlation and Spearman’s correlation (for details, see Supporting Materials and Methods). Thus, the known clinical correlation between diabetes and CVD is likely not due to altered mechanical properties of fibrin fibers as a result of hyperglycated fibrinogen. 3) The diameter of the fibrin fibers ranged from ∼20 nm to 400 nm; therefore, we also investigated the effect of diameter on single fibrin fiber mechanical properties. We observed a strong negative power law relationship between the fiber modulus, *Y*, and fiber diameter, *D*: *Y* scales as *D*^−1.6^ (plasma samples) and *D*^−1.4^ (purified fibrinogen). The strong dependence of the modulus on fiber diameter is very unusual and has interesting and significant consequences for whole-clot properties, and especially for the internal structure and lateral assembly of fibrin fibers, as discussed in the next paragraphs.

### Whole-clot modulus

Because thin fibers are stiffer, whole clots composed of many thin fibers would have a higher modulus than clots composed of fewer thick fibers, at similar fibrinogen concentrations. In a study performed with near-physiological fibrinogen concentrations (ranging from ∼1 mg/mL to 8 mg/mL), clots that formed at higher thrombin concentrations were shown to have thinner fibers (and more branch points) (4). And, indeed, the whole-clot modulus was also shown to increase in these clots with thinner fibers (4). This increase has generally been attributed to the increased density of branch points observed in clots with small-diameter fibers. However, in this study, we also observed an increased fiber modulus as fiber diameter decreased, in the absence of branch points. Thus, the increased modulus of clots formed from thinner fibers might be due to an increased modulus of the single fibers that make up the clot, as well as to increased branch points.

### Internal fibrin fiber structure and lateral fiber assembly

**Figure 6:**
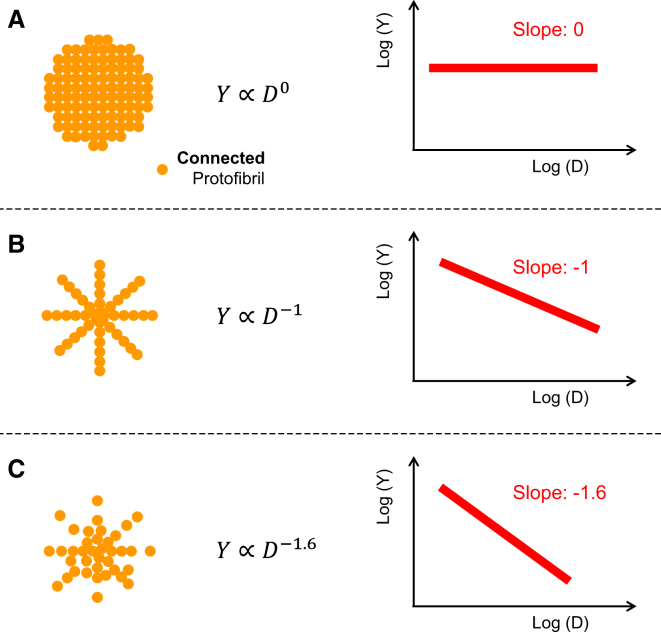
Fibrin fiber models and their corresponding stretch modulus. (*A*) A fiber with a cross section of uniformly connected protofibrils will have a stretch modulus that is independent of diameter, *D*. (*B*) A fiber with a bicycle-spokes-like cross section will have a stretch modulus that decreases as *D*^−1^. (*C*) In our experiments, the stretch modulus scales as *D*^−1.6^, indicative of a cross section in which the density of connected protofibrils likewise decreases strongly with increasing *D*, as *D*^−1.6^. To see this figure in color, go online.

The stretch modulus is a material property that is used to define the stiffness of a material under tensile stress. The modulus does not depend on the dimensions of the material (e.g., fiber diameter), provided that the material composition is homogeneous. In our fiber context, “homogeneous” means that the fiber would have a uniform density of equally connected protofibrils in the radial direction (Fig. 6 *A*). Our observation that the modulus of fibrin fibers depends on the diameter implies that fibrin fibers do not have a homogeneous cross-sectional composition. Thus, we propose, to our knowledge, a new model in which the density of the protofibrils and/or the protofibril connections within a fiber varies with diameter (Fig. 6). It is important to note that since we measure the strength of a fiber, we can draw conclusions about the density of the bonds that connect the protofibrils together, not just the density of the protofibrils.

From a mechanics of materials point of view, the Young’s modulus is proportional to the density and strength of the bonds that connect the material subunits in the longitudinal direction. For fibrin fibers, these are the bonds that connect protofibrils to each other. Since fibers grow simultaneously in the longitudinal and lateral directions to form a mature fiber, protofibrils need to assemble in a staggered fashion to form a mature fiber (28). This means that the lateral bonds between protofibrils also provide longitudinal strength. The key bonds that connect protofibrils are most likely the bonds within the network of *α*-C regions (1, 2) (Fig. 1 *B*).

As indicated in Fig. 1, we assume that fibrin fibers consist of an array of longitudinally arranged, ribbon-shaped protofibrils (28). In such an array, we further assume that protofibrils are connected with each other such that the force required to stretch a fibrin fiber is proportional to the number of bonds connecting the protofibrils. For a cross section in which the protofibrils are evenly distributed, the number of protofibril bonds, *N*_Pb_, increases proportionally to the cross-sectional area (*A* = (*π*/4)*D*^2^ for a circular cross section) and thus to *D*^2^. The force to stretch a fiber, *F*(*D*), increases as *D*^2^, and the Young’s modulus would be independent of *D* (Fig. 6 *A*). The cross-sectional protofibril bond density, *ρ*_Pb_ = *N*_Pb_/A, would be constant, i.e., independent of *D*. Using similar arguments, if we assume that a fibrin fiber forms a bicycle-spokes-like structure of protofibrils, the number of protofibril bonds per cross section, *N*_Pb_, would increase linearly with diameter. Thus, the stretching force required to reach a specific strain would also increase linearly as a function of the radius, F(D)∝D; the protofibril bond density, *ρ*_Pb_, would vary as 1/*D*; and the Young’s modulus would also vary as 1/*D* (Fig. 6 *B*). In our experiments, the Young’s modulus decreased even more strongly with diameter, as *D*^−1.6^. To explain these data, we propose a model in which the protofibril bond density, *ρ*_Pb_, also varies as *D*^−1.6^ (Fig. 6 *C*). In this model, the fiber has a dense core of well-connected protofibrils that becomes less dense as more protofibrils aggregate onto the outside of the fiber. The longitudinal cross section of such a structure is shown in Fig. 1 *C*. It may seem that the structure in Fig. 6 *C* is not stable, since protofibrils are missing at the periphery. However, it should be kept in mind that the unstructured *α*-C connector, which is part of the network of connected *α*-C domains, is 61 nm long and thus could bridge distances of tens of nanometers between inner and outer protofibrils. These connections are not shown in Fig. 6 *C*, but are schematically shown in Fig. 1 *B*. It should also be kept in mind that protofibrils are typically a few hundred nanometers long in the longitudinal direction, which means that they can form many lateral (radial) connections along their length.

We will now discuss this model in the context of Yang et al.’s (28) multibundle model, which is based on protein-protein contacts as seen in various fibrinogen crystals, and some recent diffraction, scattering, and imaging experiments that probed the internal structure of fibrin fibers. Yang et al. proposed that fibrin monomers assemble into wavy protofibril ribbons via the known and well-accepted A:a, B:b, and D:D interactions. Protofibrils then assemble via lateral associations between *γ*-chains and *β*-chains into mature fibers. It is likely that the largely unstructured *α*-C regions also play a critical role in the lateral assembly of protofibrils (1, 29). The protofibrils are staggered in the *x* and *y* (lateral) and *z* (longitudinal) directions. This stagger along all three axes is required so that fibers can grow in the lateral and longitudinal directions simultaneously. The multibundle model results in a regular crystalline fiber assembly with unit cell dimensions of 19 nm × 19 nm in the lateral (radial) dimension and 46 nm in the longitudinal dimension. Peaks corresponding to these unit cell dimensions have been observed in energy dispersive x-ray diffraction (30) and small-angle x-ray scattering (SAXS) experiments (31). However, the peaks corresponding to the lateral periodicity (19 nm) were broad and weak (31), indicating only weak ordering in the lateral (radial) direction. Moreover, AFM images suggest that the 22.5 nm periodicity (corresponding to the half-staggered arrangement of the 45 nm fibrin monomer in the longitudinal direction) disappears as fibers increase in diameter (32). SAXS and light-scattering data point to a fiber with a protein content of only 15% and a very porous cross section that becomes increasingly porous as the diameter increases (31). AFM rupture-force experiments on dry fibers also suggest a fiber cross section that becomes increasingly porous with increasing diameter (33). All of these experiments suggest a loose, open, and only weakly crystalline fiber internal structure. Some studies suggest that the density of protofibrils may decrease with increasing radius (31, 32, 33).

Our experiments probed the density and/or strength of the connections between protofibrils, and the results constitute direct evidence that the connections between protofibrils become fewer and/or weaker as the fiber diameter increases. In all of our plasma fibers, the density/strength of the protofibril connections decreased as approximately *D*^−1.6^ (Fig. 6 *C*). The underlying structural reason for this decreasing density or strength of connections between protofibrils could be 1) a decrease in the actual protein/protofibril density with increasing diameter, 2) a decrease in the number of connections between protofibrils with increasing diameter, 3) a decrease in the strength of connections between protofibrils with increasing diameter, or 4) a combination of these factors. To distinguish among these four possibilities, additional experiments that could quantitatively determine the actual protein/protofibril density as a function of fiber diameter would be needed. Possible techniques to use for such experiments could include electron microscopy of fiber cross sections, turbidity (light scattering) or SAXS experiments on fibrin clots, and fluorescence intensity measurements on single fibers. Electron microscopy could provide images of a fiber cross section (33); however, this technique has the drawback that fibers shrink as they are processed for and imaged in an electron microscope (34). Turbidity and SAXS experiments on fibrin clots indicate that larger fibers do have lower protein density (31). However, it may be difficult to extract accurate, quantitative values for the protein density in single fibers. A combined AFM/fluorescence microscope could be used to determine the fluorescence intensity of fluorescently labeled fibers as a function of diameter (33). The AFM is required to determine the diameter of the fibers, since the fibrin fiber diameter is below the resolution limit of optical microscopy. Such measurements could provide a trend of fluorescence, which scales with protein density, versus fiber diameter (33).

### Implications for fiber assembly

It is not yet clear what mechanisms might restrict bond formation between protofibrils and restrict protofibril aggregation as fibers grow thicker. One possible mechanism is twisting, which increases the path length and stretching of protofibrils in thicker fibers, i.e., protofibrils lose registry due a changed binding geometry as the fiber diameter increases (35). Another mechanism that would also be consistent with our data is activation- or diffusion-limited aggregation (36) of fibrin fibers from protofibrils. Recent work on whole clots found that incipient clots have a fractal dimension of 1.7 (37), and the authors suggested that clots may assemble via activation-limited aggregation of clusters of rod-like particles (38). When applied to the assembly of single fibers, these activation- or diffusion-limited aggregation mechanisms would result in a fractal fiber cross section in which the protofibril density would decrease with increasing diameter, as we have observed experimentally. In the context of a fiber cross section, the fractal dimension, *F*, is the exponent in the relationship between the number of protofibrils, *N*, and the diameter of the fiber, *D*: *N* = *D*^F^. For a solid, homogeneous cross section (Fig. 6 *A*), *F* would be 2. For purely diffusion-limited aggregation, *F* would be ∼1.7, i.e., the fiber’s protein content would decrease as its diameter increased. For activation- and diffusion-limited aggregation, *F* would be less than that. In our data (Fig. 6 *C*), the bond density, *ρ*_Pb_, scales as *D*^−1.6^, and thus the number of bonds per cross section, *N*_pb_, would scale as *D*^–1.6 + 2^ = *D*^0.4^_._ *N*_pb_ = *ρ* × *A*, where *A* is the cross-sectional area of the fiber, *A* = *π* × (*D*/2)^2^. Thus, our data suggest that fibrin fibers have a very low fractal dimension of 0.4 for the number of bonds in a fibrin fiber cross section. Our data suggest that fibrin fibers start out with a well-connected semicrystalline core of protofibrils, but then the fiber becomes increasingly porous and disorganized as more protofibrils aggregate. This increased disorganization may be consistent with the out-of-registry assembly model (35) and the recently proposed early-branching model (39).

### Clinical implications and fibrinolysis

There is increasing experimental and clinical evidence that fibrin clots composed of highly branched networks with thin fibers are associated with thrombosis, in particular myocardial infarction, ischemic stroke, and venous thromboembolism (5, 6, 7, 8). There is also experimental evidence that clots with thinner fibers lyse more slowly than clots with thick fibers, even when normalized for total fibrinogen concentration (40). Our model provides a simple explanation for the slower lysis of clots composed of thin fibers: thin fibers have a higher bond density than thick fibers and thus are harder to lyse.

### Blood clot modeling

Modeling of whole clots can help to reveal correlations among clot mechanical properties, diseases, and treatment. It should be possible to calculate the bulk mechanical properties of a whole fibrin clot by knowing the properties of the individual components. Our single-fiber data provide the foundation for determining initial parameters for modeling whole fibrin clots. A distinct advantage of modeling with correct single-fiber properties is that it enables one to determine the differences in the mechanical properties of networks with very dissimilar structures. Our results demonstrate why it is important to measure the properties of different types of individual fibers before incorporating them into a model.

In previous work, we reported the average modulus of fibrin fibers (11, 41) and did not take the currently observed diameter dependence of the modulus into account. This means that the modulus of thin fibers was underestimated and the modulus of thick fibers was overestimated. Typically, the diameter of the previously examined fibers was on the order of 100–160 nm, and the over- and underestimates approximately balanced out. Differences observed in comparing two types of fibers, e.g., cross-linked and uncross-linked fibers, will still hold up if the diameter distribution of the samples is approximately the same. This was the case in all of our work. The diameter dependence of the modulus will have the biggest impact for samples that are made up of thinner or thicker than normal fibers.

## Conclusions

Using plasma samples from uncontrolled and controlled diabetic individuals and a nondiabetic control group, we determined that glycation does not seem to have an effect on single fibrin fiber mechanical properties. This implies that the observed epidemiological correlation between diabetes and CVD likely does not have a molecular origin at the single fibrin fiber level.

Using these plasma samples and samples prepared from purified fibrinogen, we observed a strong dependence of fibrin fiber modulus on fiber diameter, *D*: the modulus decreases as *D*^−1.6^. This observation can be best explained with a new fibrin fiber model in which the cross-sectional density of bonds within fibrin fibers decreases with increasing diameter; that is, fibrin fibers become less densely connected as their diameter increases. Such a model is not consistent with a crystalline, homogeneous cross section of equally connected protofibrils, where the modulus would be independent of *D*.

Our findings imply that any parameter that affects the diameter of fibrin fibers, such as the thrombin concentration, will have a strong effect on the modulus of single fibers. This in turn will have a strong effect on whole-clot mechanical properties and, presumably, the in vivo behavior of blood clots. Clinically, our model provides a simple explanation for the observation that clots composed of thin fibers are harder to lyse: thin fibers have a higher bond density than thick fibers. In addition, in clot dissolution, lytic factors (e.g., plasminogen) and plasminogen activators (e.g., tPA, urokinase, and streptokinase) need to reach the inside of clots and fibrin fibers (see plasmin cleavage sites in Fig. 1 *A*). Our model, in which thinner fibers are denser than thick fibers, implies that it should be easier to dissolve clots that consist of fewer thick fibers than those that consist of many thin fibers, which is consistent with experimental (40) and clinical (7) observations.

## Author Contributions

W.L., J.S., and C.H. collected data, designed experiments, and wrote/edited the manuscript. M.P. provided the diabetes samples, designed experiments, and wrote/edited the manuscript. C.N. provided microscopy images and analysis. J.W. aided in the research design and edited the manuscript. M.G. designed experiments, wrote/edited the manuscript, and was the overall supervisor of this project.

## Supporting Material

Document S1. Supporting Materials and Methods and Figs. S1–S6

Document S2. Article plus Supporting Material
