# Supplementary material for: Fibrin Fiber Stiffness Is Strongly Affected by Fiber Diameter, but Not by Fibrinogen Glycation
Source: Biophys J. 2016 Mar 29;110(6):1400–10. doi: 10.1016/j.bpj.2016.02.021 (PMC4816776; doi:10.1016/j.bpj.2016.02.021)
Supplement: Document S1. Supporting Materials and Methods and Figs. S1–S6 [file mmc1.pdf]

Biophysical Journal, Volume *110*

Supplemental Information

Fibrin Fiber Stiffness Is Strongly Affected by Fiber Diameter, but Not by Fibrinogen Glycation

Wei Li, Justin Sigley, Marlien Pieters, Christine Carlisle Helms, Chandrasekaran Nagaswami, John W. Weisel, and Martin Guthold

SUPPLEMENT

Incremental stress-strain curves

Figure S2 shows an example of an incremental stress-strain curve to determine the moduli and stress relaxation times. The fiber is stretched then held at a constant strain for a period of time before being pulled again. The process is repeated at higher and higher strains. The plots show that as a fiber is held at constant strain, the stress decays. This is indicative of viscoelastic behavior. The simplest mechanical model that can account for these observations (the two relaxation rates; stress does not decay to zero) is a generalized Kelvin model (Figure S1), consisting of an elastic spring with modulus Y_∞, in parallel with two Maxwell elements consisting of a dashpot and a spring in series (1). For this model, the equation for stress relaxation becomes

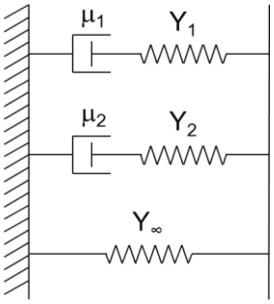

Figure S1. *Generalized Kelvin Model*

$$\sigma(t) = \varepsilon_0 \left[Y_\infty + Y_1 \cdot e^{-t/\tau_1} + Y_2 \cdot e^{-t/\tau_2} \right] \quad (1).$$

Y_∞ is the relaxed elastic modulus, Y is the total elastic modulus, $Y = Y_\infty + Y_1 + Y_2$, and ε_0 is the strain at which the fiber is held. By fitting an double-exponential curve to the stress decay (Figure S3), we can determine key mechanical properties – the total modulus, the elastic modulus, and relaxation times of the fibers (1). A single exponential does not fit well to the stress relaxation curves (Figure S3).

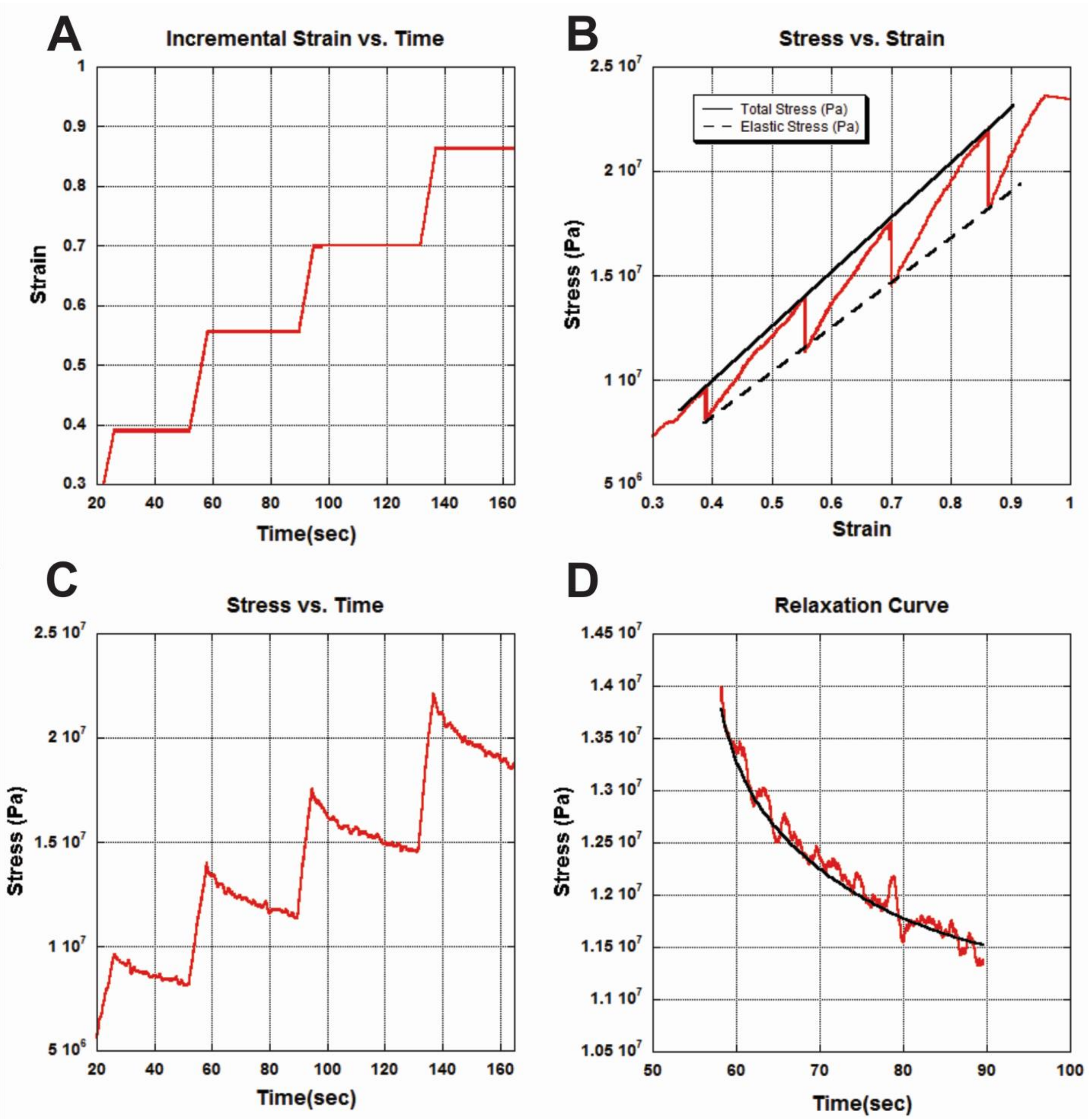

Figure S2. Incremental stress–strain curves and relaxation. (A) Strain versus time for an incrementally stretched fiber. The fiber was stretched to a given length and held at a constant strain while allowing the fiber to relax. The plateaus on the graph indicate where the AFM tip was stopped and the fiber was held at constant strain during the manipulation. (B) Stress versus strain for fibrin fiber that was pulled incrementally. The red curve is the raw data for the fiber, the black solid line approximates the total modulus determined by the stress before relaxation, and the black dashed line approximates the elastic modulus component of the total modulus. Exact values were

determined by fitting the data with equation 1. (C) Stress versus time for an incrementally stretched fiber. Stress decays exponentially when the fiber is held at a constant strain, indicating viscoelastic behavior. (D) Stress relaxation curve for fibrin fiber. The curve was fit with a double exponential function (equation 1) which produced two relaxation times. The fast relaxation time for this fiber was τ_1 = 2.1 s and the slow relaxation time was τ_2 = 17.8s.

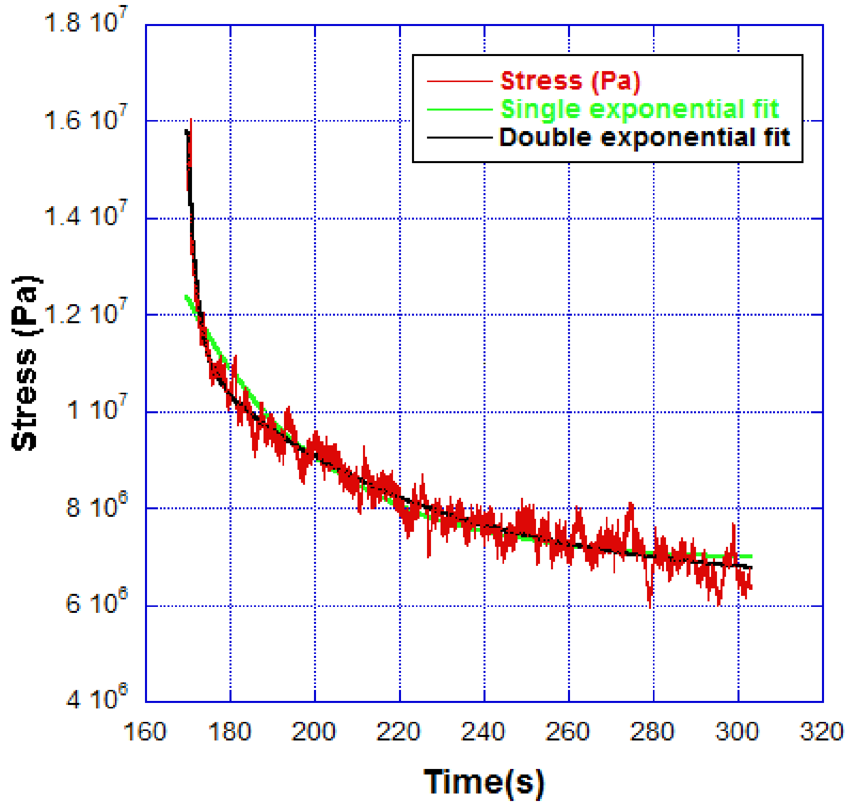

Figure S3. Single exponential and double exponential fit to incremental stress-strain curve.

<u>Relaxed Elastic Modulus</u>

In addition to the total stretch modulus Y, which we report in the main manuscript, we also determined the relaxed, elastic modulus, Y_∞, from the fits of the incremental stress strain curves.

For all experiments, we found that elastic modulus Y_∞ is typically about a factor of 0.6 smaller than the total stretch modulus, Y, and that it shows the same behavior as Y. In particular, Y_∞ does not depend on glycation and fibrinogen concentration, like Y. Y_∞ also shows a similar diameter dependence as Y. Y_∞ also decreases with increasing diameter, as $Y_\infty \propto D^{-1.5}$. The data for Y_∞ are summarized in supplemental figure S4.

Elastic modulus (Relaxed Modulus) vs. Glycation

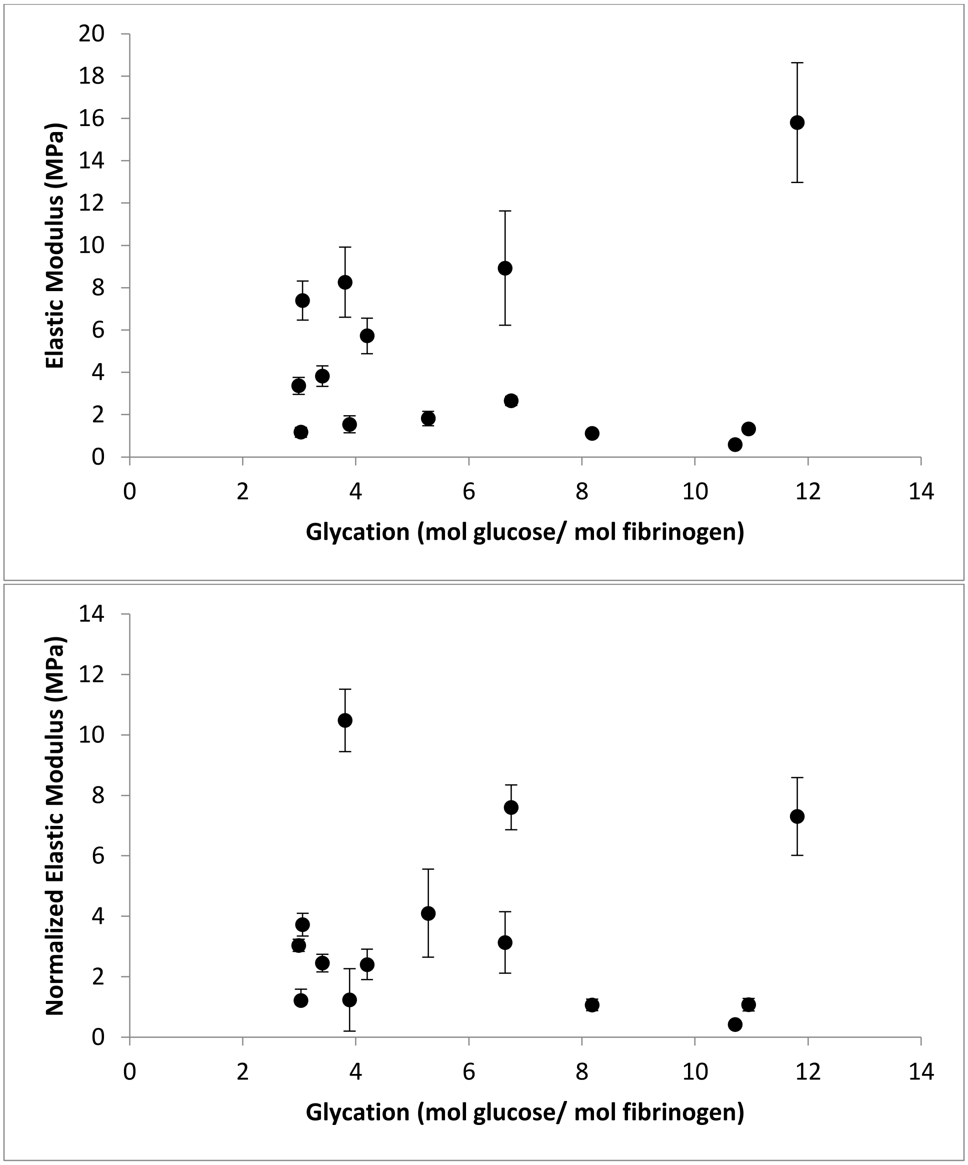

Elastic modulus (Relaxed Modulus) vs. Fibrinogen

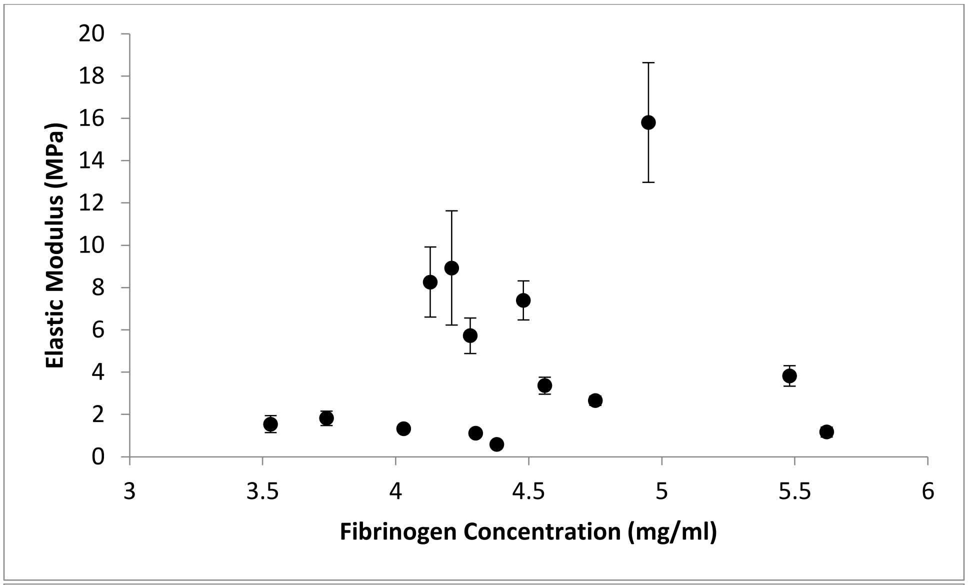

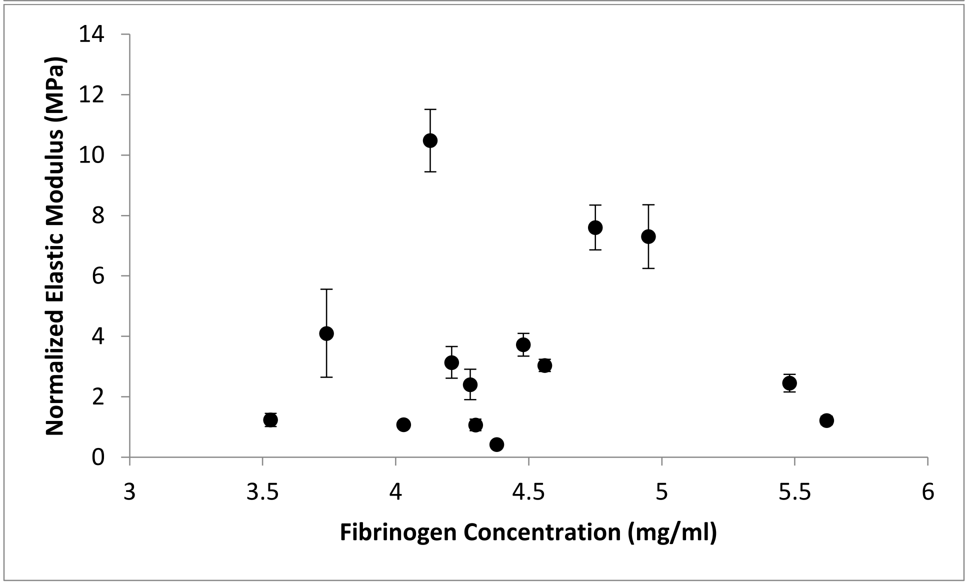

Elastic modulus (Relaxed Modulus) vs. Fibrin Fiber Diamter

Plasma sample

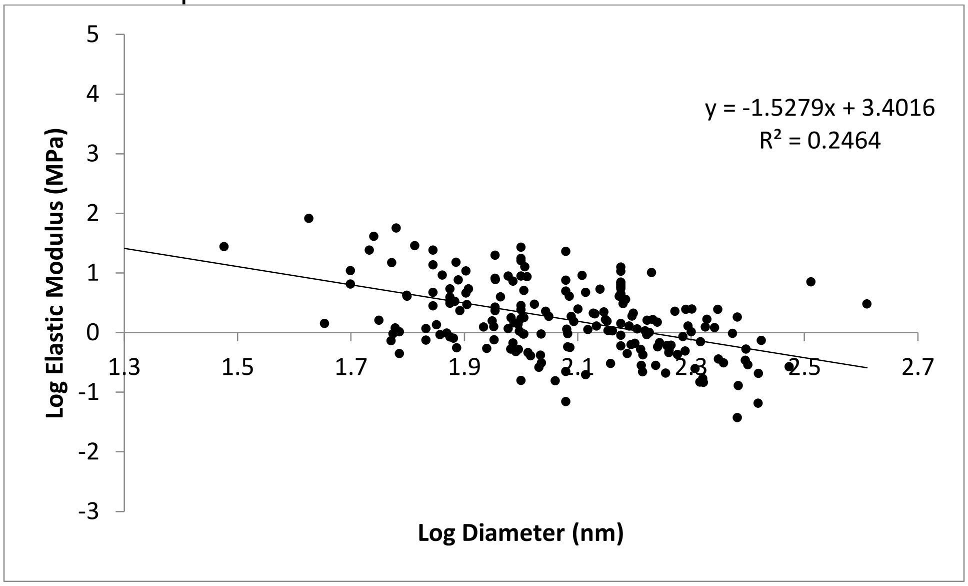

Purified Fibrinogen sample

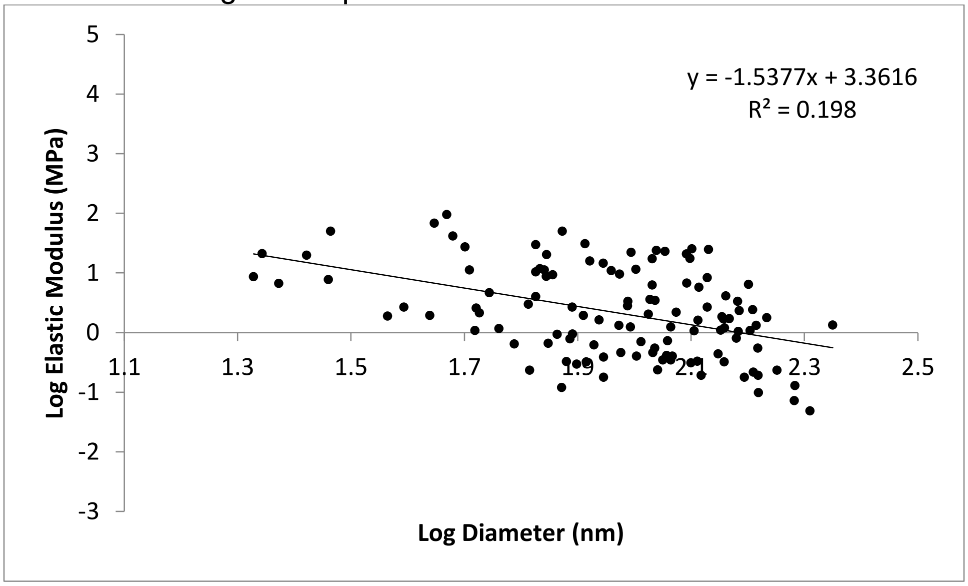

Figure S4 (previous three pages). The relaxed, elastic modulus Y_∞ as a function of glycation (no dependence), fibrinogen concentration (no dependence), and diameter (strong $D^{-1.5}$ dependence).

Complete set of single fiber parameters extracted from generalized Kelvin model

	E_0 (MPa)	E_∞ (MPa)	τ_1 (s)	τ_2 (s)	E_1 (MPa)	E_2 (MPa)	μ_1 (MPa*s)	μ_2 (Mpa*s)
**Uncontrolled diabetes**	9.4 ± 1.8	5.6 ± 1.2	2.5 ± 0.6	34 ± 3	0.23 ± 0.06	1.53 ± 0.39	0.6 ± 0.2	52 ± 16.
**Controlled diabetes**	13 ± 2	4.6 ± 0.7	2.3 ± 0.3	31 ± 2	0.29 ± 0.04	3.42 ± 1.15	0.7 ± 0.2	106 ± 40
**Healthy control group**	5.3 ± 1.2	3.1 ± 0.5	2.0 ± 0.2	31 ± 3	0.66 ± 0.12	9.41 ± 2.33	1.3 ± 0.3	295 ± 93
**Total plasma sample**	9.5 ± 1.4	4.5 ± 0.8	2.3 ± 0.4	32 ± 3	0.35 ± 0.07	4.0 ± 1.1	0.8 ± 0.3	127 ± 41

All the samples are from South African black females aged 40-65 years.

<u>Scanning Electron Microscopy</u>

Scanning electron microscopy was used to determine the fibrin structure of clots formed from purified fibrinogen of diabetic and control subjects. Clots were formed by addition of 0.5U/ml α-human thrombin and 3mM $CaCl_2$ to 1mg/ml fibrinogen in 0.15M NaCl, 0.05M Tris-HCl, pH 7.4 (final concentrations). Samples were prepared as described previously (2). Clots were observed and photographed digitally in many different areas, using a scanning electron microscope (XL 20,

FEI, Hillsboro, Oregon, USA). Fiber diameters were measured from micrographs at 10,000 x magnification using ImageJ software (National Institutes of Health, USA). The thicknesses of at least 100 different fibers were measured per micrograph, with at least 6 micrographs imaged for each patient. Using scanning electron microscopy (SEM) on the plasma samples, we found that the average fibrin fiber diameter slightly decreases from about 105 nm to 85 nm (from fitted line) as the *original plasma* fibrinogen concentration increases from about 3.5 mg/ml to 5.5 mg/ml (Supplementary Figure S5). However the samples investigated by SEM were all formed from purified fibrinogen and investigated at 1 mg/ml.

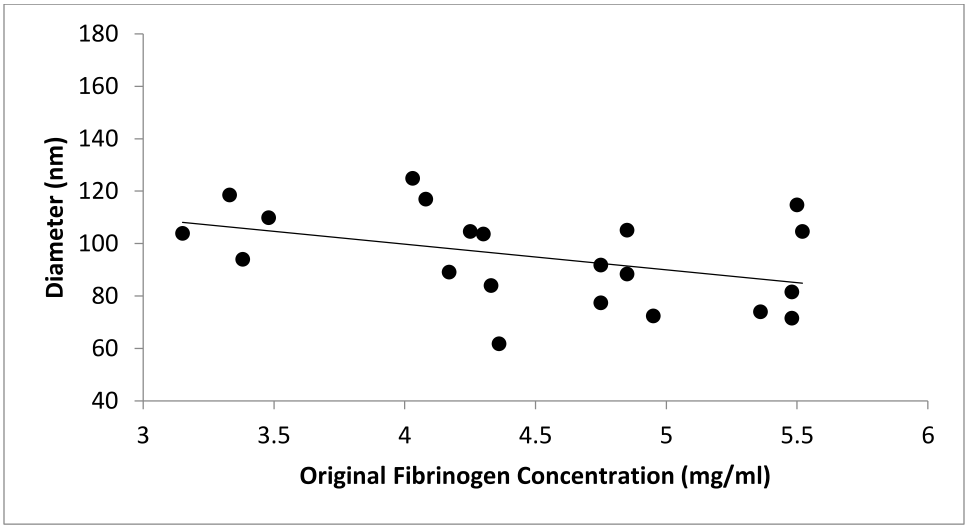

Figure S5. Fiber diameter, as determined by SEM (N = 21, R^2 = 0.1731), slightly decreases with increasing *original plasma* fibrinogen concentration. However, all SEM measurements were done with fibrinogen at 1 mg/ml that was purified from the original plasma samples.

Diameter distribution

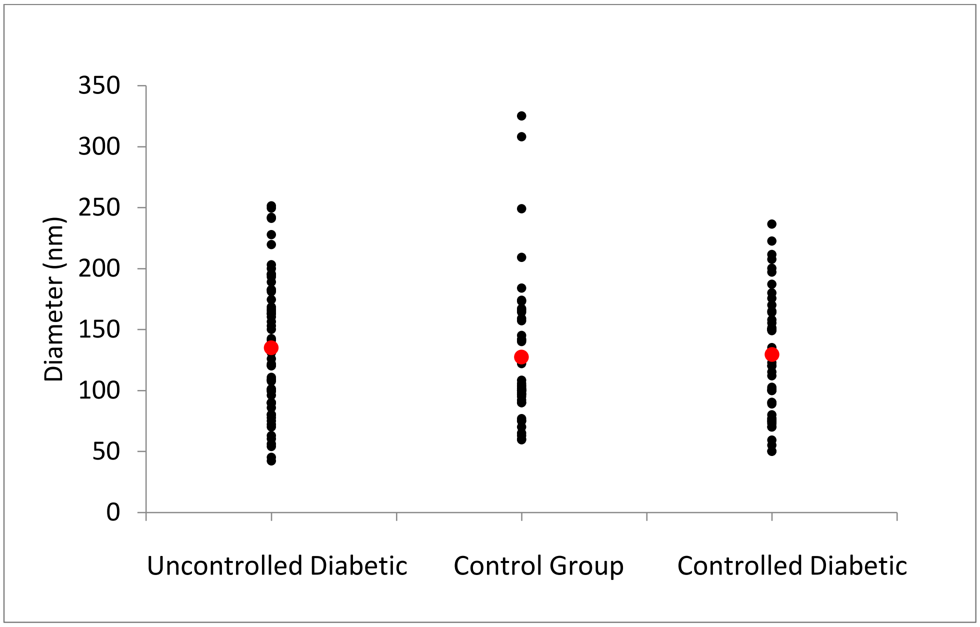

Figure S6. Fiber diameter distributions for each group. The red dot represents the average diameter, D_{avg}, for each group; uncontrolled diabetic, D_{avg} = 130 ± 4 nm (N = 60); control group, D_{avg} = 130 ± 1 nm (N = 56), controlled diabetic, D_{avg} = 130 ± 3 nm (N = 45).

Statistical Analysis

We use Pearson's correlation (testing linear relation) and Spearman's correlation (testing monotonic relation) to test if there is relationship between two variables. Detail are in the following table.

	Pearson's correlation	Spearman's correlation
Glycation vs. Total Modulus	-0.028	-0.007
Glycation vs. Normalized Total Modulus	-0.133	-0.174
Fibrinogen Concentration vs. Total Modulus	0.065	0.046
Fibrinogen Concentration vs. Normalized Total Modulus	0.018	0.103
Glycation vs. Extensibility	-0.072	-0.095
Fibrinogen Concentration vs. Extensibility	0.161	0.143
Glycation vs. Fast Relaxation Time	0.258	0.018
Glycation vs. Slow Relaxation Time	-0.065	-0.042
Fibrinogen Concentration vs. Fast Relaxation Time	-0.229	-0.418*
Fibrinogen Concentration vs. Slow Relaxation Time	-0.251	-0.151

	Pearson's correlation
Diameter vs. Total Modulus (Plasma sample)	-0.513
Diameter vs. Total Modulus (Purified Fibrinogen)	-0.405

Pearson's correlation is testing linear relationship. A value between 0.00 and 0.19 (-0.00 to -0.19) means "no or negligible relationship". A value between -0.4 and -0.69 means "strong negative relationship"

Spearman's correlation is testing if one variable is monotonic function of the other. Again, a value between 0.00 to 0.19 means "very weak relation"

There is “no or negligible relationship’ between any of the mechanical properties and glycation and fibrinogen concentration. The one exception is the Spearman’s correlation value of “Fibrinogen concentrate vs. Fast Relaxation Time” (labeled with a *), which indicates a monotonic relationship. However, the Pearson’s correlation value for this pair is small (indicating no/weak linear relationship). Moreover, when inspecting the plot, there also is no clear relationship. We, therefore, believe there also is no significant relationship between fibrinogen concentration and fast relaxation time.

Both tests show a strong, negative correlation between fiber modulus and diameter.

References

1. Liu W, Carlisle CR, Sparks EA, & Guthold M (2010) The Mechanical Properties of Single Fibrin Fibers. *Journal of Thrombosis and Haemostasis* 8(5):1030-1036.
2. Pieters M, *et al.* (2008) Glycaemic control improves fibrin network characteristics in type 2 diabetes - A purified fibrinogen model. *Thrombosis and Haemostasis* 99(4):691-700.
